# Cyanobacteria: A Precious Bio-resource in Agriculture, Ecosystem, and Environmental Sustainability

**DOI:** 10.3389/fmicb.2016.00529

**Published:** 2016-04-21

**Authors:** Jay Shankar Singh, Arun Kumar, Amar N. Rai, Devendra P. Singh

**Affiliations:** ^1^Department of Environmental Microbiology, Babasaheb Bhimrao Ambedkar UniversityLucknow, India; ^2^Department of Biochemistry, North-Eastern Hill UniversityShillong, India; ^3^Department of Environmental Science, Babasaheb Bhimrao Ambedkar UniversityLucknow, India

**Keywords:** agriculture, bioremediation, beneficial microbes, bio-fertilizers, cyanobacteria

## Abstract

Keeping in view, the challenges concerning agro-ecosystem and environment, the recent developments in biotechnology offers a more reliable approach to address the food security for future generations and also resolve the complex environmental problems. Several unique features of cyanobacteria such as oxygenic photosynthesis, high biomass yield, growth on non-arable lands and a wide variety of water sources (contaminated and polluted waters), generation of useful by-products and bio-fuels, enhancing the soil fertility and reducing green house gas emissions, have collectively offered these bio-agents as the precious bio-resource for sustainable development. Cyanobacterial biomass is the effective bio-fertilizer source to improve soil physico-chemical characteristics such as water-holding capacity and mineral nutrient status of the degraded lands. The unique characteristics of cyanobacteria include their ubiquity presence, short generation time and capability to fix the atmospheric N_2_. Similar to other prokaryotic bacteria, the cyanobacteria are increasingly applied as bio-inoculants for improving soil fertility and environmental quality. Genetically engineered cyanobacteria have been devised with the novel genes for the production of a number of bio-fuels such as bio-diesel, bio-hydrogen, bio-methane, synga, and therefore, open new avenues for the generation of bio-fuels in the economically sustainable manner. This review is an effort to enlist the valuable information about the qualities of cyanobacteria and their potential role in solving the agricultural and environmental problems for the future welfare of the planet.

## Introduction

The present world population of about 7.2 billion is expected to cross 9.6 billion by the end of year 2050. In order to provide food to all by that times, the annual production of cereals needs a jump of about 50%, i.e., from 2.1 billion tons per year to ∼3 billion tons per year. This onerous target puts enormous pressure on agriculture sector to achieve the food security. But such a quantum leap in food production can be achieved either by bringing more and more land under cultivation or by enhancing the productivity of cultivable land available. The first option remains a distant dream in the light of limited land and growing population. The option of increasing soil fertility and agricultural productivity with the help of better eco-friendly management tools, promises a successful food security.

The current agricultural practices are heavily dependent on the application of synthetic fertilizers and pesticides, intensive tillage, and over irrigation, which have undoubtedly helped many developing countries to meet the food requirement of their people; nevertheless raised environmental and health problems, which include deterioration of soil fertility, overuse of land and water resources, polluted environment, and increased cost of agricultural production. A big question before the present day agriculture is to enhance the agricultural production to meet the present and future food requirements of the population within the available limited resources, without deteriorating the environmental quality (Singh and Strong, 2016). The sustainable agriculture practices can fulfill the growing need of food as well as environmental quality (Mason, 2003). The present philosophy of sustainable agriculture includes eco-friendly, low-cost farming with the help of native microorganisms. It also emphasizes that the farmers should work with natural processes to conserve resource such as soil and water, whilst minimizing the cost of agricultural production and waste generation that adversely affects the environment quality. Such sustainable agricultural management practices will make the agro-ecosystem more resilient, self-regulating and also maintain the productivity and profitability.

Since long, the microbes have been known to contribute to the soil fertility and sustainable green energy production (Koller et al., 2012). During the last decades, the microbial processes of green energy production have gained interest as the sustainable tool for the generation of bio-fuels, namely methane (CH_4_), ethanol, H_2_, butanol, syngas, etc. Current investigations witnessed noteworthy surge growth in the production of cyanobacterial biomass for bio-fuels, food supplements (super foods), and bio-fertilizers for safe agriculture (Yamaguchi, 1997; Benson et al., 2014). They have been classified as beneficial as well as harmless bio-agents based on their role in regulating plant productivity. In reality, these two diverse groups of microorganisms coexist in nature, and predominance of one at any point of time, depends mainly on the environmental conditions. For many years, soil microbiologists and microbial ecologists have been studying the effect of beneficial or efficient soil microorganisms for sustainable agriculture which not only contribute to soil fertility, crop growth and yield, but also improve the environment quality.

**FIGURE 1:**
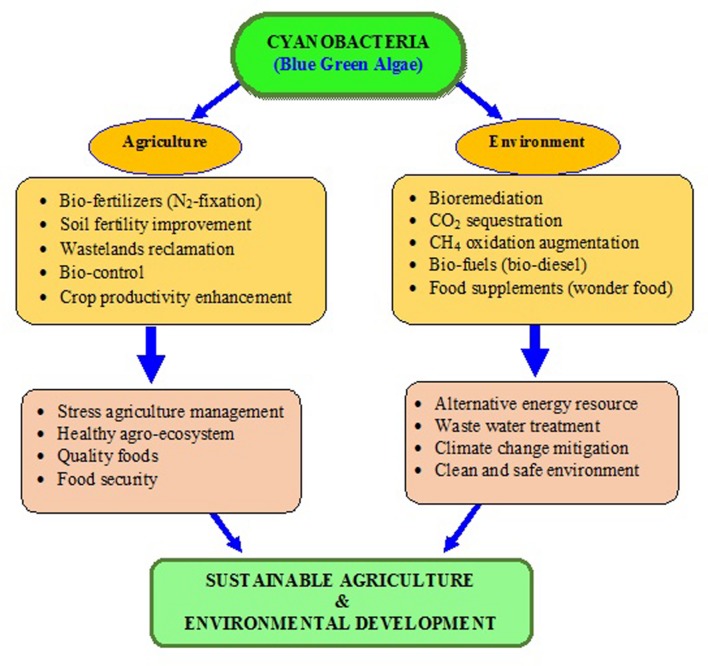
**A hypothetical model exhibiting the potential roles of cyanobacteria in sustainable agriculture and environmental management**.

Nowadays, sustainable agriculture practices have envisaged an important role of these tiny microorganisms in achieving the food security without creating environmental problems. The recent trends of using the bio-inoculants containing beneficial soil microbes over synthetic fertilizers, insecticides, and pesticides for enhancing crop productivity is a welcome step. As a beneficial microbe, cyanobacteria could play a potential role in the enhancement of agriculture productivity and mitigation of GHG emissions (Singh, 2011; Singh et al., 2011a). Very recently, it has been proposed that cyanobacteria could be the vital bio-agents in ecological restoration of degraded lands (Singh, 2014). Cyanobacteria are the group of photosynthetic organisms which can easily survive on bare minimum requirement of light, carbon dioxide (CO_2_) and water (Woese, 1987; Castenholz, 2001). They are phototrophic, and naturally occur in several agro-ecosystems like paddy fields and from Antarctica to Arctic poles (Pandey et al., 2004). They fulfill their own nitrogen requirement by nitrogen (N_2_)-fixation, and produce some bioactive compounds, which promote the crop growth/protect them from pathogens and improve the soil nutrient status. Cyanobacteria are also useful for wastewater treatment, and have the ability to degrade the various toxic compounds even the pesticides (Cohen, 2006). A conceptual model about the role of cyanobacteria in sustainable agriculture and environmental management has been proposed (**Figure 1**). This review highlights the role of cyanobacteria in bio-energy production, ecological restoration, agriculture and environmental sustainability.

## Methods

**FIGURE 2:**
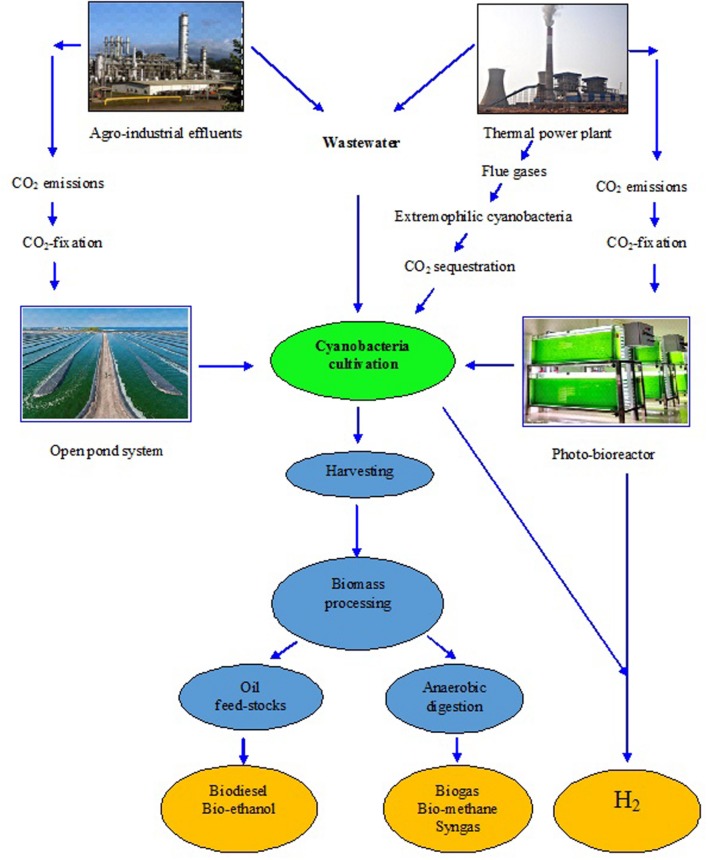
**A model showing the technologies used for production of bio-fuels from the mass cultivation of cyanobacteria**.

**FIGURE 3:**
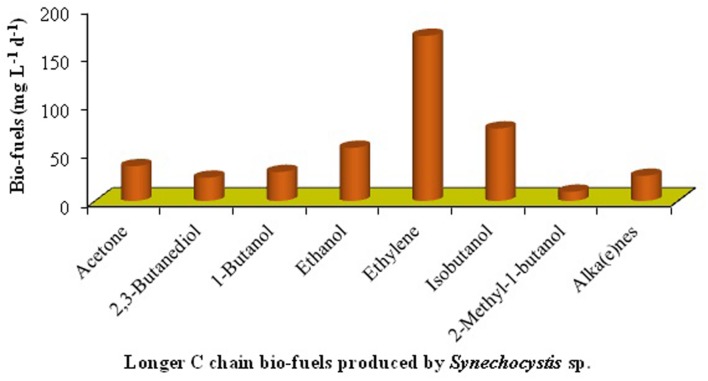
**Production of bio-fuels from genetically modified cyanobacteria (data has been modified from Machado and Atsumi, 2012; Nozzi et al., 2013)**.

The reviews by the authors covered the valuable role of cyanobacteria in development of sustainable agriculture and environment is described in **Figures 1** and **2**, with different approaches proposed. In this article, the present viewpoint of sustainable agriculture and environment includes eco-friendly, low-cost farming with the help of bio-agents like cyanobacteria based on specific, internally reliable hypothesis and values. The various approaches proposed in **Figures 1–3** demonstrate that cyanobacteria are the effective tool for enhancing the soil fertility, bio-fuel production, bioremediation, reducing GHGs emissions, and enhancing crop productivity. Finally, it was proposed that the genetically engineered cyanobacteria can be exploited as the multi-functional bio-agents for eco-friendly agriculture and other beneficial uses for sustainable development (**Tables 1–7**).

**Table 1:** Important nitrogen fixing cyanobacterial genera.

Form of Cyanobacteria	Cyanobacterial members
Unicellular	*Aphanothece, Chroococcidiopsis, Dermocapsa, Synechococcus, Gloeocapsa* (*Gloeothece*)^∗^*, Myxosarcina, Pleurocapsa*^∗^*, Xenococcus*
Filamentous heterocystous	*Anabaena*^∗^*, Anabaenopsis, Aulosira, Calothrix*^∗^*, Camptylonema, Chlorogloea, Chlorogloeopsis, Cylindrospermum, Fischerella*^∗^*, Gloeotrichia, Hapalosiphon, Mastigocladus, Nodularia, Nostoc*^∗^*, Nostochopsis, Rivularia, Scytonema*^∗^*, Scytonematopsis, Stigonema, Tolypothrix, Westiella, Westiellopsis*
Filamentous non-heterocystous	*Lyngbya, Microcoleus chthonoplastes, Myxosarcina, Oscillatoria, Plectonema boryanum, Pseudanabaena, Schizothrix, Trichodesmium*

*^∗^Some strains of these genera live symbiotically with other plants (Sinha and Häder, 1996)*.

**Table 2:** Cyanobacterial species exhibiting antagonistic effects against different plant pathogens.

Cyanobacteria	Plant diseases and pathogens	Reference
*Calothrix elenkenii*	Damping off (*Rhizoctonia solani*)	Manjunath et al., 2009
*Fischerella muscicola*	Brown rust (*Uromyces appendiculatus*), powdery mildew (*Erysiphe graminis*), rice blast (*Pyricularia oryzae*)	Hagmann and Juttner, 1996
*Nostoc muscorum*	Cottony rot of vegetables and flowers (*Sclerotinia sclerotiorum*) and damping off (*Rhizoctonia solani*)	De Caire et al., 1990; Kulik, 1995; Tassara et al., 2008


**Table 3:** Heavy metal removal by some cyanobacterial species.

Heavy metals	Source	Cyanobacteria	Reference
Cd	Sewage water aqueous solution	*Nostoc linckia, N. rivularis, Tolypothrix tenuis*	Inthorn et al., 1996; El-Enany and Issa, 2000
Co	Sewage and industrial wastewater	*N. muscorum, Anabaena subcylindrica*	El-Sheekh et al., 2005
Cr	Metal contaminated soil	*N. calcicola, Chroococcus* sp.	Anjana et al., 2007
Cu	Sewage and industrial wastewater	*N. muscorum, A. subcylindrica*	El-Sheekh et al., 2005
Hg	Wet biomass	*Spirulina platensis, Aphanothece flocculosa*	Cain et al., 2008
Mn	Sewage and industrial wastewater	*N. muscorum, A. subcylindrica*	El-Sheekh et al., 2005
Pb	Sewage and industrial wastewater	*N. muscorum, A. subcylindrica, Gloeocapsa* sp.	El-Sheekh et al., 2005; Raungsomboon et al., 2006
Zn	Sewage water	*N. linckia, N. rivularis*	El-Enany and Issa, 2000

**Table 4:** An overview of production of plant growth promoting chemicals by cyanobacteria.

Type	Cyanobacteria	Reference
Auxins	*Anabaena, Anabaenopsis, Calothrix, Chlorogloeopsis, Cylindrospermum, Glactothece, Nostoc, Plactonema, Synechocystis*, etc.	Ahmad and Winter, 1968; Mohan and Mukherji, 1978; Selykh and Semenova, 2000; Sergeeva et al., 2002
Gibberellins	*Anabaenopsis, Cylindromum*, etc.	Singh and Trehan, 1973; Mohan and Mukherji, 1978
Cytokinins	*Anabaena, Chlorogloeopsis, Calothrix*, etc.	Rodgers et al., 1979; Selykh and Semenova, 2000

**Table 5:** Some cyanobacterial members and their lipid contents (modified from Sharathchandra and Rajashekhar, 2011).

Cyanobacteria	Lipid contents (%)	Lipid types
*Calothrix fusca*	22.60	Palmitic acid, linoleic acid
*Lyngbya dendrobia*	10.55	Palmitic acid, palmitoleic acid, oleic acid, linoleic acid
*L. limnetica*	26.45	Palmitic acid, oleic acid, linoleic acid
*Microcystis aeruginosa*	28.15	Tridecanoic acid, palmitic acid, stearic acid, linoleic acid, α-linolenic acid
*Nostoc linckia*	18.45	Palmitic acid, oleic acid, linoleic acid
*Oscillatoria calcuttensis*	25.70	Lauric acid, palmitic acid, palmitoleic acid, Heptadecanoic acid, stearic acid, oleic acid, linoleic acid
*O. acuminata*	24.65	Lauric acid, tridecanoic acid, myristic acid, palmitic acid, palmitoleic acid, heptadecanoic acid, stearic acid, oleic acid, linoleic acid
*O. chlorina*	16.62	Palmitic acid, stearic acid, oleic acid, linoleic acid, lignoceric acid
*O. amoena*	18.63	Palmitic acid, oleic acid, linoleic acid, lignoceric acid
*O. perornata*	14.10	Palmitic acid, palmitoleic acid
*Phormidium ambiguum*	10.48	Lauric acid, palmitic acid, lignoceric acid
*Scytonema bohnerii*	22.22	Palmitic acid, linoleic acid

**Table 6:** Chemical products synthesized by genetically engineered cyanobacteria.

Product	Cyanobacteria members	Reference
1-Butanol Fatty acids Isoprene Isobutyraldehyde Isobutanol	*Synechococcus elongatus* PCC7942 *Synechocystis* sp. PCC 6803 *Synechocystis* sp. PCC 6803 *S. elongatus* PCC 7942 *S. elongatus* PCC 7942	Lan and Liao, 2011 Liu et al., 2011 Lindberg et al., 2010 Atsumi et al., 2009 Atsumi et al., 2009

**Table 7:** Bio-methane producing cyanobacterial members.

Cyanobacteria	C/N Ratio	Methane yield	Reference
*Arthrospira maxima* *A. platensis* *Microcystis* sp. *Spirulina* Leb18 *Spirulina* sp. *S. platensis* UTEX1926	4.3–5.33 – – – 4.16 –	173 mL g^-1^ 481 mL g^-1^ 70.33–153.51 mL 0.79 g L^-1^ 0.35–0.80 m^3^ 0.40 m^3^	Inglesby and Fisher, 2012 Mussgnug et al., 2010 Zeng et al., 2010 Costa et al., 2008 Samson and Leduy, 1986 Converti et al., 2009

## Cyanobacteria in Sustainable Management

Beneficial microbes are an alternative to other management practices. The cyanobacteria are bestowed with ability to fix atmospheric N_2_, decompose the organic wastes and residues, detoxify heavy metals, pesticides, and other xenobiotics, catalyze the nutrient cycling, suppress growth of pathogenic microorganisms in soil and water, and also produce some bioactive compounds such as vitamins, hormones, and enzymes which contribute to plant growth (Higa, 1991). These bio-agents can improve the soil quality and plant growth, and minimize the crop production cost by supplementing the good crop management practices such as crop rotation, use of organic manures, minimum tillage, and the bio-control of pests and diseases. The use of cyanobacteria in agriculture promises definite beneficial effects on crop productivity, if used properly (Higa and Wididana, 1991).

The currently used traditional agriculture management practices heavily rely on the application of chemical fertilizers and pesticides, and practices like intensive tillage and excess irrigation which otherwise lead to ever increasing cost of agricultural production, over exploitation of natural resources like soil and water, and also create environmental pollution (Kumar et al., 2012). Now, there is need to adopt such sustainable agricultural practices which are not only eco-friendly, but are also cost-effective, and really help us attain the long-term food security. Some of the major objectives of sustainable agriculture include production of safe and healthy foods, conservation of natural resources, economic viability, restoration and conservation of ecosystem services. An eco-friendly management approach for complex agro-ecosystem without disturbing the interactions among number of ecological components like water, edaphic and climatic factors including the living components offers the long-term rise for sustainable increase in productivity. It may be suggested that if the four major ecosystems processes, i.e., energy flow, water cycle, mineral cycles, and ecosystem dynamics, function together without disturbing the harmony or homeostasis of individual components, can ultimately reduce the cost of agriculture production.

The application of cyanobacteria in management of soil and environment includes the economic benefits (reduced input cost), nutrient cycling, N_2_-fixation, bioavailability of phosphorus, water storage and movement, environmental protection and prevention of pollution and land degradation especially through reducing the use of agro-chemicals, and recycling of nutrients and restoration of soil fertility through reclamation (Shukia et al., 2008).

The following benefits to the agro-ecosystem are offered through use of cyanobacteria:

• Enhanced solubilization and mobility of nutrients of limited supply.
• Complexing of heavy metals and xenobiotics to limit their mobility and transport in plants.
• Mineralization of simpler organic molecules such as amino acids for direct uptake.
• Protection of plants from pathogenic insects and diseases as bio-control agents.
• Stimulation of the plant growth due to their plant growth promoting attributes.
• Improving the physico-chemical conditions of soils.

### Cyanobacteria under Extreme Environments

Cyanobacteria commonly known as blue-green-algae, are not truly eukaryotic algae. They are Gram-negative prokaryotes, perform oxygenic photosynthesis, and also fix atmospheric N_2_. They are ubiquitous in ponds, lakes, water streams, rivers, and wetlands. They can easily survive the extreme environments such as hot springs, hyper-saline waters, freezing environments, and arid deserts (Singh, 2014). Cyanobacteria are able to survive at a temperature range of 45–70°C (Castenholz, 1978) and pH lower than 4–5 (Pfennig, 1969, 1974) with optimum range of 7.5–10 (Fogg, 1956). The ability of cyanobacteria to survive extreme environmental conditions can be exploited for amelioration of the salt affected soils as they can reduce the salt content and promote levels of C, N, and P including moisture content of the salt affected soils. It has been noticed that cyanobacteria induces soil aggregation and water permeability, and are quite useful in improving quality of poor structured soils of arid or sub-arid areas. Rogers and Burns (1994) investigated that inoculation of cyanobacteria enhanced the stability of soil aggregate (important characteristics of good soil and noticed the resistance of aggregates to wetting and physical disruption); that improved WHC and aeration in soils. Such organisms reduce the compaction and sodicity of soils through improvement in the level of organic carbon, WHC, aeration and support the biodiversity of other microflora.

### Cyanobacteria as Bio-fertilizers

Cyanobacteria fix atmospheric N_2_ by forms, i.e., free-living and symbiotic associations with partners such as water fern *Azolla*, cycads, *Gunnera*, etc. A list of free-living and symbiotic N_2_ fixing cyanobacteria has been described in **Table 1**. Some cyanobacterial members are endowed with the specialized cells known as heterocyst – thick-walled modified cells, which are considered site of nitrogen fixation by nitrogenase enzyme. The enzyme is a complex, catalyzes the conversion of the molecular N_2_ into reduced form like ammonia (Singh et al., 2011). The fixed nitrogen may be released in the form of ammonia, polypeptides, free amino acids, vitamins, and auxin-like substances; either by secretion or by microbial degradation after the cell death (Subramanian and Sundaram, 1986). Nitrogen-fixing ability has not only been shown by heterocystous cyanobacteria but also by several non-heterocystous unicellular and filamentous genera (**Table 1**). Cyanobacteria can contribute to about 20–30 kg N ha^-1^ as well as the organic matter to the soil, quite significant for the economically weak farmers unable to invest for costly chemical nitrogen fertilizer (Issa et al., 2014). There is a little knowledge on commercial byproducts or biofertilizers but several cyanobacterial species such as *Anabaena variabilis, Nostoc muscorum, Aulosira fertissima*, and *Tolypothrix tenuis* found to be effective biofertilizers. Many Asian countries like China, Vietnam, India, etc., have been utilizing cyanobacteria in paddy cultivation as the alternative to nitrogen fertilizers (Venkataraman, 1972; Lumpkin and Plucknett, 1982). It has been reported that N availability to plants is increased due to application of cyanobacteria in agriculture ecosystems, particularly the rice fields (Stewart et al., 1968; Peters et al., 1977; Singh and Singh, 1987). Several researchers have investigated that inoculation of cyanobacteria (*in vitro*) in wheat crops, could enhance the plant shoot/root length, dry weight, and yield (Spiller and Gunasekaran, 1990; Obreht et al., 1993; Karthikeyan et al., 2007, 2009), but the agronomic efficiency has not been evaluated (Gantar et al., 1991, 1995a,b; Kaushik, 2012).

It has also been suggested that cyanobacteria can improve the bioavailability of phosphorus to the plants by solubilizing and mobilizing the insoluble organic phosphates present in the soil with the help of phosphatase enzymes. Cyanobacteria have the ability to solubilize the insoluble form of (Ca)_3_(PO_4_)_2_, FePO_4,_ AlPO_4_, and hydroxyapatite [Ca_5_(PO_4_)_3_OH] in soils and sediments (Bose et al., 1971; Dorich et al., 1985; Wolf et al., 1985; Cameron and Julian, 1988). There are two hypotheses regarding the mechanism of solubilization of phosphate by cyanobacteria;

(a) Cyanobacteria synthesize a chelator for Ca^2+^ which drives the dissolution to the right without changing the pH of growth medium (Cameron and Julian, 1988; Roychoudhury and Kaushik, 1989) as mentioned below-

$${Ca}_{10}{\left(OH\right)}_{2}{\left({PO}_{4}\right)}_{6}\to {10Ca}^{2+}+{2OH}^{-}+{{6PO}_{4}}^{3-}$$

(b) The other assumption is that cyanobacteria release organic acids, which can solubilize phosphorus through following reaction (Bose et al., 1971) as given below-

$${Ca}_{3}{\left({PO}_{4}\right)}_{2}+{2H}_{2}{CO}_{3}\to {2CaHPO}_{4}+{Ca\left({HCO}_{3}\right)}_{2}$$

Besides the above said two mechanisms, there is also a third possibility. Once an inorganic phosphate is solubilized, the resulting PO_4_^3-^ is scavenged by the growing population of cyanobacteria for their own nutrition needs, and after their death, of the cell locked PO_4_^3-^ released in the soils, is easily available to plants and other organisms following mineralization (Arora, 1969; Saha and Mandal, 1979; Mandal et al., 1992, 1999).

Fuller and Roger (1952) observed that uptake of phosphorus by plants from algal materials was greater than that from the inorganic phosphates, when both were provided in equal amounts over a longer period of time. They also proposed the hypothesis that cyanobacteria could remove available phosphorus from the sphere of chemical fixation in soil by incorporating it into cell constituents or by absorbing it in excess amounts, and then releasing it gradually for plants over a period of time through exudation, autolysis or microbial decomposition of dead cells.

### Cyanobacteria as Bio-control Agents

The antagonistic effects of cyanobacteria against different plant diseases have been presented in **Table 2**. Cyanobacteria produce a variety of biologically active compounds of antibacterial, antifungal, antialgal, and antiviral potential (Teuscher et al., 1992; Dahms et al., 2006). These bioactive compounds belong to the group of polyketides, amides, alkaloids, fatty acids, indoles, and lipopeptides (Abarzua et al., 1999; Burja et al., 2001). In addition cyanobacteria produce a broad spectrum of anti-algal compounds which inhibit growth of pathogens by disturbing their metabolic and physiological activities (Dahms et al., 2006).

The cell constituents of cyanobacteria are known to reduce the incidence of *Botrytis cinerea* on strawberries and *Erysiphe polygoni* causing powdery mildew on turnips and damping off disease in tomato seedlings, besides reducing the growth of saprophytes—*Chaetomium globosum*, *Cunninghamella blakesleeana*, and *Aspergillus oryzae*, and plant pathogens such as *Rhizoctonia solani* and *Sclerotinia sclerotiorum* (Kulik, 1995). Several researchers reported that compounds like Fischerellin from *Fischerella muscicola*, shows antifungal activity against several plant pathogenic fungi such as *Uromyces appendiculatus* (brown rust), *Erysiphe graminis* (powdery mildew), *Phytophthora infestans* and *Pyricularia oryzae* (rice blast), but it was less effective against *Monilinia fructigena* (brown rot) and *Pseudocercosporella herpotrichoides* (stem break; Hagmann and Juttner, 1996; Papke et al., 1997).

Among cyanobacteria, *Nostoc muscorum* has been shown to be antifungal against soil fungi and especially those producing “damping off” (De Caire et al., 1990). The fungus *Sclerotinia sclerotiorum*, causes “white mold,” one of the most polytheist plant pathogens, mostly affecting compositae notably lettuce (*Lactuca sativa* L.) and other species of rosette plants (Tassara et al., 2008). Extracts from *N. muscorum* inhibited the *in vitro* growth of the fungal plant pathogens such as *S. sclerotiorum* (Cottony rot of vegetables and flowers) and *Rhizoctonia solani* (root and stem rots; Kulik, 1995). Biondi et al. (2004) reported that *Nostoc* sp., a known potential cryptophycin producer, is the source of natural pesticides against the fungi, insects, nematodes. Zulpa et al. (2003) ascertained that *N. muscorum* also inhibited the growth of other fungi producing the “wood blue stain” [bluish or grayish discoloration of sapwood caused by certain dark-color fungi (*Aureobasidium, Alternaria, Cladosporium*, etc.)] on the surface and in the interior of the wood (Zulpa et al., 2003). It seems that efficient cyanobacterial strains can be used as bio-control agents to secure higher agriculture yield. New assays are using the cyanobacterial metabolites for obtaining commercial products for sustainable agriculture development. However, information about the bio-controls shows most experiments have been conducted under lab conditions, and very few in the natural agriculture fields. Therefore, there is need for an extensive research to find out the feasibility to apply cyanobacteria as the potential bio-control agents against various plant diseases.

### Cyanobacteria in Reclamation of Salt Affected Soils

Cyanobacteria could be playing a potential role in the reclamation of salt affected (generally termed as Usar land in some parts of India), arid or sub-arid soils. For amelioration of salt affected lands, chemical methods of using gypsum, sulfur or excessive irrigation applied (Dhar and Mukherji, 1936), are not so cost-effective or environment friendly. Basically, salt affected soils (alfisol/sodic/alkaline/saline) are less productive, rigid soils impermeable to water due to the presence of excessive salts in the upper layers. They can be classified as alkaline and/or saline depending on the salt content. The alkaline soil is characterized by a high pH, high exchangeable Na, measurable amounts of carbonates, and it undergoes extensive clay dispersion (deflocculation due to the high zeta potential of active Na^+^). The poor hydraulic conductivity and reduced soil aeration make the soils infertile. The saline soil is characterized by high amount of soluble salts (electrical conductivity more than 4 dS cm^-1^), imparting high osmotic tension to plant roots for absorption of water and nutrients (Pandey et al., 1992). For the first time, Singh (1961) suggested that cyanobacteria could be used as tool for reclamation of Usar soils as they form a thick stratum on the soil surface and conserve the organic C, N, and P as well as moisture, and convert the Na^+^ clay to Ca^2+^ clay. Organic matter and N added by the cyanobacteria in such soils helps binding of the soil particles and thus, improves soil permeability and aeration (Singh, 1961). Since the cyanobacteria are capable of solubilizing nutrients from insoluble carbonate nodules through the secretion of oxalic acid (Fritsch, 1945; Singh, 1961); they improve the physico-chemical quality of saline and alkali soils such as soil aggregation by lowering the pH, electrical conductivity, and hydraulic conductivity (Kaushik and Subhashini, 1985). There are certain physiological advantages associated with cyanobacteria which enable them withstand these stresses:

(a) Curtailment of Na^+^ influx (Apte et al., 1987)
(b) Accumulation of inorganic (K^+^ ion) or organic osmoregulators (sugars, quaternary amines, etc.; Reed et al., 1984)

Cyanobacterial application to organically poor semi-arid soils can play a significant role in their reclamation. The soils in these deserts or semi-arid regions are characterized high compaction, low fertility, and water deficiency; and also associated with problems of salinity and sodicity (Nisha et al., 2007); result in poor aeration and water infiltration, more soil erosion, and poor diversity of micro-flora. The poor physico-chemical characteristic of soils ultimately has an adverse impact on the plant growth and productivity. Cyanobacteria develop a superficial network of the trichomes/filaments on the soil, which not only binds the soil particles, but also result in enmeshing of the soil particles at depth (Nisha et al., 2007). Cyanobacteria, as carbon and nitrogen fixers, can contribute to the improvement of soil nutrient status of organic carbon and nitrogen in arid soils. Cyanobacterial species such as *Anabaena oscillarioides, A. aphanizomenoides*, and *Microcystis aeruginosa* exhibited the salt tolerance ability ranging from 7 to 15 g/L (Coutinho and Seeliger, 1984; Moisander et al., 2002).

They are also known for the production of EPS, which help soil particles to bind together (Mazor et al., 1996), and thus play a major role in improvement of soil moisture owing to their hygroscopic nature. Flaibani et al. (1989) reported that exopolysaccharides from cyanobacteria also contribute to reclamation of the desert soils.

### Cyanobacteria in Bioremediation

Cyanobacteria as bioremediators, have some advantages over other microorganisms because of their photoautotrophic nature and ability to fix atmospheric N_2_ which makes them self-sufficient for growth and maintenance and adaptability to survive in polluted and heavily polluted environments (Sokhoh et al., 1992). Cyanobacteria show a great potential for the treatment of various types of environmental contaminates such as pesticides (Megharaj et al., 1994), crude oil (Sokhoh et al., 1992; Al-Hasan et al., 1998, 2001), naphthalene (Cerniglia et al., 1980a,b), phenanthrene (Narro et al., 1992), phenol and catechol (Shashirekha et al., 1997), heavy metals (Singh et al., 2011b), and xenobiotics (Megharaj et al., 1987) either through their accumulation or degradation. Due to high metal sorption capacity and high multiplication rate, cyanobacteria could play a potential role in the detoxification of various industrial eﬄuents such as from oil refinery, brewery and distilleries, paper mill, sugar mill, dye and pharmaceuticals industries. Cyanobacteria may be used for tertiary treatment of urban, agro-industrial eﬄuents (Vílchez et al., 1997), and in turn, can help mitigate eutrophication and metal toxicity problems in aquatic ecosystems. Because of their photosynthetic nature, some cyanobacterial species are conferred with the additional advantages like higher interior pH, which is almost two units higher than the surrounding pH conditions and this confers resistance to mass transfer of pollutants out of their biofilms from the external environment and thus helps in removal of heavy metals from the wastewaters (Liehr et al., 1994; Vijayakumar, 2012). Currently, cyanobacteria have been used efficiently as low-cost bioremediating agents for treatment of N-, P-rich dairy wastewaters and converting these nutrients into biomass (Lincoln et al., 1996; Singh et al., 2011a). Cyanobacteria accumulate very high concentration of pesticides (Vijayakumar, 2012). Cyanobacterial members such as *Synechococcus elongatus*, *Anacystis nidulans*, and *Microcystis aeruginosa* degrade many organo-phosphorus and organo-chlorine insecticides from the polluted aquatic systems (Vijayakumar, 2012). El-Bestawy et al. (2007) reported that several cyanobacterial genera *Oscillatoria*, *Synechococcus*, *Nodularia*, *Nostoc*, *Microcystis*, and *Anabaena* have the ability to remove or degrade the lindane residues. According to Forlani et al. (2008), cyanobacteria like *Anabaena* sp., *Lyngbya* sp., *Microcystis* sp., and *Nostoc* sp. degrade the broad range organo-phosphorous herbicide glyphosate, and the mineralized glyphosate, is consumed as the phosphorus source. Lipok et al. (2007, 2009) demonstrated that *Spirulina* sp. could degrade the glyphosate herbicide. It is also reported that *Synechocystis* sp. successfully mineralized the aniliofos herbicide, and used the product as phosphate source. Thus it is evident that the cultivation of cyanobacteria in wastewater lagoons may have great potential to degrade the pollutants and pesticides, and help in reducing the pollution load and support growth of other microbial populations for reductions in the BOD and COD.

Several investigations showed that cyanobacteria degrade crude oil and other complex organic compounds such as surfactants (Radwan and Al-Hasan, 2000; Raghukumar et al., 2001; Mansy and El-Bestway, 2002). For example, cyanobacterial species *Oscillatoria salina, Plectonema terebrans, Aphanocapsa* sp., and *Synechococcus* sp., develop mats in aquatic environments, and have been successfully used in the bioremediation of oil spills in different parts of the world (Raghukumar et al., 2001; Cohen, 2002). Not only oil-contaminated waters but also oil-contaminated soils be successfully remediated using a naturally occurring cyanobacterial–bacterial associations (Sorkhoh et al., 1995). Al-Hasan et al. (1998) reported that *Microcoleus chthonoplastes* and *Phormidium corium* isolated from oil-rich sediments of the Arabian Gulf, were able to degrade *n*-alkanes. It is also reported that *Oscillatoria* sp. and *Agmenellum* sp. oxidize naphthalene to 1-naphthol (Cerniglia et al., 1979, 1980a); *Oscillatoria* sp. oxidize biphenyl to 4-hydroxybiphenyl (Cerniglia et al., 1980b) and *Agmenellum* sp. metabolizes phenanthrene into *trans*-9,10-dihydroxy-9,10-dihydrophenanthrene, and 1-methoxy-phenenthrene (Narro, 1985). The role of cyanobacterial species in removal of heavy metals in different ecosystems is given in the **Table 3**. The biodegradation potential of cyanobacteria can be enhanced through genetic engineering (Kuritz and Wolk, 1995), and be used as the economical and maintenance-free remediation technology for contaminated eco-systems. However, ecological and environmental concerns and regulatory constraints are the major obstacles for releasing the transgenic cyanobacteria for bioremediation purposes under field conditions. Since most of the research on bioremediation by engineered cyanobacteria is of basic nature, there is growing need for the regulatory, security or economical-beneficial systems, which may decipher their bioremediation potential tool into reality.

### Cyanobacteria as Plant Growth Promoters

Cyanobacteria release extracellular plant growth promoting substances; some described as hormones (**Table 3**) like gibberellins (Singh and Trehan, 1973), cytokinin (Rodgers et al., 1979), auxin (Ahmad and Winter, 1968), or abscisic acids (Marsalek et al., 1992). Others are explained as vitamins, particularly vitamin B (Grieco and Desrochers, 1978) or amino acids (Vorontsova et al., 1988), antibiotics and toxins. The production of phytohormones by some potential cyanobacteria is outlined in **Table 4**.

Most of the studies on the plant growth promoting effects of cyanobacteria related to paddy crop revealed that cyanobacterial inoculation could enhance rice seed germination, root and shoot growth (Misra and Kaushik, 1989a,b). It is also evident that co-inoculation of cyanobacteria with wheat enhanced root dry weight and chlorophyll (Obreht et al., 1993). Gantar et al. (1995a,b) observed that extracellular substances released by cyanobacteria that colonize wheat plant roots showed significant effect on plant growth, though the agronomic efficiency was not evaluated. Due to their natural diversity, the capacity of cyanobacteria to grow in a variety of locations, even those unfit for agriculture, could be exploited. The fast cyanobacterial cell growth and simple nutritional requirements mainly water, sunlight, and CO_2_ (Ruffing, 2011) provides a wide scope for the commercial application of cyanobacterial species as plant growth promoters.

### Cyanobacteria as Source of Bio-energy

**FIGURE 4:**
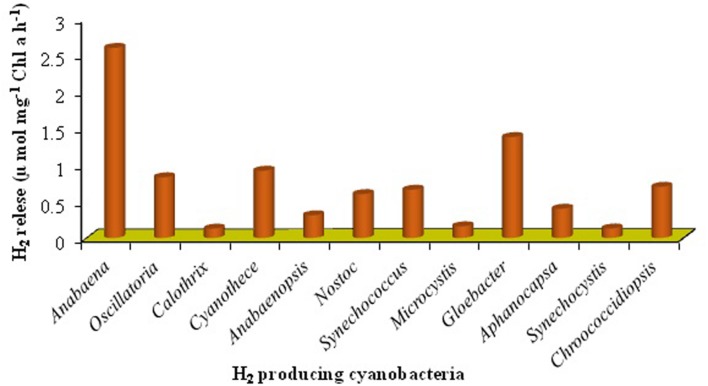
**Production of H_2_ from cyanobacteria (data has been modified from Dutta et al., 2005)**.

**FIGURE 5:**
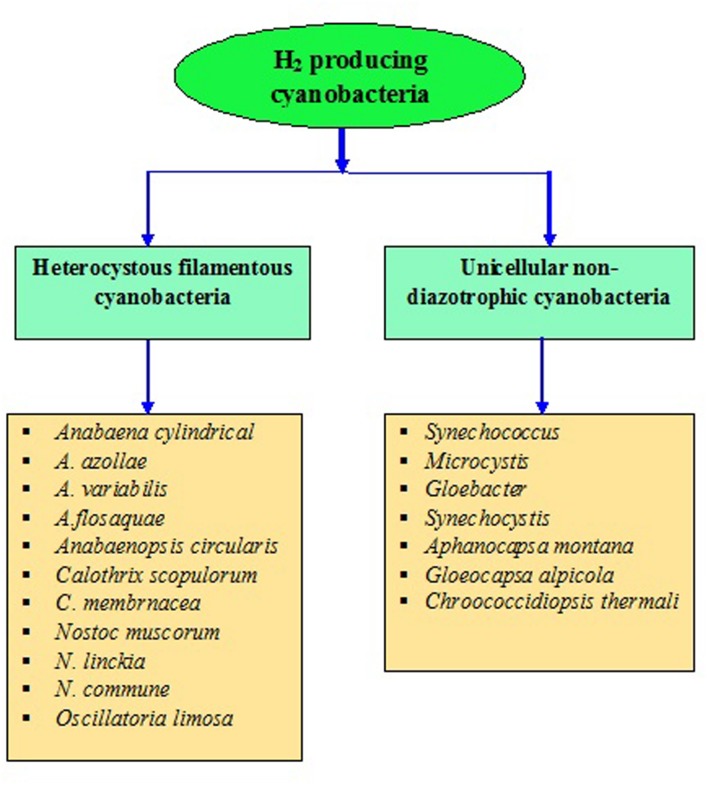
**Heterocystous and non-diazotrophic H_2_ producing cyanobacteria**.

Cyanobacteria are the unique group of photosynthetic bio-agents that can grow at a fast rate due to their simple cell structure and minimum requirement of nutrients accompanied by the capacity to produce bio-energy including bio-diesel, bio- or syngas, bio-hydrogen, etc. (Kumar and Singh, 2016) (**Figure 2**). Carbon dioxide (CO_2_) is taken up by cyanobacteria through photosynthesis, to be converted to carbon-rich lipids (**Table 5**) that can be used in production of bio-fuels. Cyanobacteria also produce molecular hydrogen (H_2_) which could be the better option and the ideal substitute of for fossil fuels. These microbes can produce different feed-stock for energy generation like H_2_ (by photosynthesis), lipids for biodiesel, jet fuel and hydrocarbons, isoprenoids for gasoline and carbohydrates for ethanol production (Parmar et al., 2011; Rosgaard et al., 2012). The cyanobacterial biomass containing lignocellulosic compounds can also be processed for syngas production followed or not by a Fischer–Tropsch process and gasification/liquefaction for H_2_ production (Lawson et al., 2011). The advantage of using molecular H_2_ as the clean fuel is one of the most abundant elements in the universe, and has maximum energy per unit weight (122 KJ g^-1^). On a weight basis, it is calculated that the heating value of H_2_ is 141.65 MJ Kg^-1^, which is the highest amongst known fuels (Ali and Basit, 1993). It can be stored as gas-metal hydride or as liquid, and has greater energy conversion efficiency than petroleum. H_2_, if used as a fuel, will not cause environmental pollution because its only by-product is water. Several cyanobacterial genera including *Anabaena, Calothrix, Oscillatoria, Cyanothece, Nostoc, Synechococcus, Microcystis, Gloeobacter, Aphanocapsa, Chroococcidiopsis*, and *Microcoleus* are known for their ability to produce H_2_ under various culture conditions (Masukawa et al., 2001; Parmar et al., 2011; Nozzi et al., 2013) (**Figures 4** and **5**). Cyanobacteria produce H_2_ by two ways (Pinzon-Gamez et al., 2005):

(a) Mediated by the nitrogenase enzyme which catalyzes the following reaction

$$N_{2}+{8H}^{+}+{8e}^{-}+16ATP\to {2NH}_{3}+H_{2}+16ADP+16Pi$$

(b) Reversible activity of hydrogenase enzymes

$$H_{2}⇌{2H}^{+}+{2e}^{-}$$

Although cyanobacterial H_2_ production is a clean and green technology, the limitation in this process is lesser H_2_ production that makes unsuitable for being economically feasible (Tiwari and Pandey, 2012). There are certain shortcomings associated with these processes which form an obstacle in the scale up of H_2_ production from cyanobacteria. The hydrogenase enzyme responsible for H_2_ production is extremely sensitive to O_2_, and therefore, the concurrent production of O_2_ poses a serious limitation. The process through which H_2_ is produced by cyanobacteria, has its merits and de-merits both in terms of technology and productivity. Undoubtedly, based on the research reports, it may be deduced that this field is yet preliminary and without any potential practical application. These processes are yet to be evaluated and modified for productivity and cost the of H_2_ commercialization.

Besides production of bio-fuel and molecular H_2_, the cyanobacterial biomass can be used to produce biogas via anaerobic digestion or fermentation (**Table 7**). The organic biopolymers (carbohydrates, lipids, and proteins), in the cyanobacterial biomass are hydrolyzed and broken down into monomers, which are then subjected to anaerobic digestion to produce biogas (mixture of CH_4_ and CO_2_). During biogas production, CO_2_ is the second main component (approximately 25–50%), which can be removed to obtain bio-methane. The calorific value of biogas can be significantly enhanced by removing the CO_2_ during bio-methane production (Hankamer et al., 2007). CH_4_ or bio-methane can be used as the compressed natural gas in vehicles, which is going to be more environmentally friendly than the fossil fuels like gasoline/petrol and diesel. Converti et al. (2009) reported biogas production and purification using a two-step bench-scale biological system, consisting of fed-batch pulse-feeding anaerobic digestion of mixed sludge, followed by CH_4_ enrichment of biogas by the use of the cyanobacteria like *Arthrospira platensis*. The ratio of CH_4_ and CO_2_ ranges between 70.5–76.0% and 13.2–19.5%, respectively. The data on CO_2_ removal from biogas revealed the existence of a linear relationship between the rates of *A. platensis* growth and CO_2_ removal from biogas, and this allows the estimation of carbon utilization efficiency of cyanobacterial biomass to the extent of almost 95% (Converti et al., 2009).

The C/N ratio is one of the important factors influencing CH_4_ production during the anaerobic digestion (Zhong et al., 2012). The high protein content of the cyanobacterial biomass (low C/N ratio) compared to terrestrial plants leads to a high ammonia release during anaerobic digestion, thus inhibiting the anaerobic micro-flora responsible for CH_4_ production (Sialve et al., 2009). Anaerobic digestion of the protein rich cyanobacterium *Spirulina maxima*, containing up to 60–71% of proteins, releases an extremely high concentration of ammonia (up to 7000 mgL^-1^). The methanogens are perhaps among the most sensitive micro-flora to high NH_3_. It is worth noting that methanogenic bacteria can, however, acclimate to high concentrations of ammonium Sialve et al. (2009). It is suggested that a significant increase in CH_4_ production can be achieved by adding carbon-rich corn straw to the co-digestion process with cyanobacterial biomass (Sialve et al., 2009). According to their result output the C/N ratio of 20/1 was found to be the best in terms of CH_4_ productivity, which increased by 61.69% during the study as compared to control. Therefore, it may be recommended that co-digestion of cyanobacterial biomass containing high protein contents (low C/N ratio) with plant residues (low protein contents or high C/N ratio) could be one of the options for efficient CH_4_ production and waste treatment.

The cultivation of these green bio-agents (cyanobacteria farming) can efficiently be done at different scales, lesser space, time and under diverse conditions (fresh as well as waste and unused waters) to achieve high value bio-fuel products. The filamentous cyanobacteria could be beneficial since contaminated and wastewaters may be used for large scale biomass production and at the same time, treating wastewater to remove pollutants. The cyanobacterial biomass quality and quantity can be manipulated with the help of several physico-chemical treatments to achieve the desired cyanobacterial biomass having good quality bio-fuel products. Bio-fuel production using cyanobacteria farming offers various advantages over other bio-agents may be:

• Fast growth and multiplication capability of cyanobacteria can meet huge demand for bio-fuels using limited resources.
• Cyanobacterial cultivation consumes less fresh water than the croplands, and wastewaters can equally be utilized for the generation of biomass.
• Under elevated CO_2_ concentrations, cyanobacterial biomass can be produced at higher efficiency.
• Green house gases (nitrous oxide, CH_4,_ etc.) emissions from crop fields can be minimized through cultivation of cyanobacteria for bio-energy production, and
• Cyanobacteria farming for generation of bio-fuels may be potentially more cost-effective, eco-friendly, and sustainable than the conventional agriculture farming.

It seems that genetically engineered cyanobacteria can be potentially used for the production of a number of bio-fuels [acetone, butanol, ethanol, alka(e)nes, etc.] in the economically sustainable way (**Figure 3** and **Table 6**). However, different biotechnological, environmental and economic challenges have to be overcome before energy products from recombinant cyanobacteria (Apt and Behrens, 1999). Further, both the production technology and downstream processing of the end products can effectively be improved to obtain super quality bio-fuels from cyanobacteria.

### Cyanobacteria in CO_2_ Sequestration and Climate Change Mitigation

Carbon dioxide is one of the purported GHGs, primarily responsible for global warming and needs to be mitigated. The strategies to reduce CO_2_ emissions include energy savings, development of renewable bio-fuels, and CCS. CCS, a viable tool needs to be explored to enhance the efficiency of such a strategy (Rau et al., 2007), and several approaches being considered are (a) capture of point-source CO_2_ from power plants or other industrial sources and subsequent injection of the concentrated CO_2_ underground or into the ocean (Benson and Orr, 2008); (b) expansion of biological carbon sequestration of atmospheric CO_2_ by measures such as reforestation, changes in land use practices, increased carbon allocation to underground biomass, production of biochar and enhanced bio-mineralization (Jansson et al., 2010).

The CO_2_ sequestration by cyanobacteria is receiving increased awareness in alleviating the influence of rising CO_2_ concentrations in the atmosphere (Kumar et al., 2011). Being photosynthetic, cyanobacteria contribute to a large share of the total photosynthetic conversion of solar energy and assimilation of CO_2_. The CO_2_ fixation rate in cyanobacteria is about 10–50 times faster than the terrestrial plants. Thus the use of these biological agents is considered one of the effective approaches to reduce the concentration of atmospheric CO_2_ and thereby, to help in mitigation of possible global warming (Chisti, 2007). The captured CO_2_ in the cyanobacterial biomass can be stored in the form of organic molecules, which can then be used in various ways. In paddy field soils, the cyanobacteria contribute significantly to both organic and nitrogenous contents (Singh, 2014).

It is anticipated that half of global photosynthesis is contributed by phytoplankton, which mostly includes cyanobacterial members (Fuhrman, 2003). Among these, about 25% of the total global photosynthesis can be accounted for by only two efficient marine cyanobacterial genera, *Synechococcus* and *Prochlorococcus* (Rohwer and Thurber, 2009). Many cyanobacteria are halophilic and, therefore, they can be cultured in marine waters, saline drainage water, or brines from petroleum refining industry or CO_2_ injection sites, thereby sparing freshwater supplies (Jansson and Northen, 2010). Combustion of fossil fuels such as coal, oil, gas, etc. is the major source of flue gas (mixture of N_2_, CO_2_, O_2_, and water vapors) emission globally. Since the flue gas released from the power plants contains high concentrations of CO_2_ and has high temperatures (about 120°C), the cyanobacterial bio-fixation of CO_2_ may warrants the use of thermophilic cyanobacterial species that are tolerant to both high CO_2_ and temperature (Badger and Price, 2003; Ono and Cuello, 2007). Biomass production and CO_2_ uptake in cyanobacteria exposed to higher CO_2_ levels from flue gas or other streams have been followed for a variety of cyanobacterial species such as *Aphanothece microscopica* (Jacob-Lopes et al., 2008). Several thermophilic cyanobacterial members (*Synechococcus aquatilis*, *Chlorogloeopsis* sp. etc.) having the capability to tolerate higher temperatures, can be used for CO_2_ sequestration from the flue gas. Though the major problem associated with the cyanobacterial or biological use of CO_2_ is the high temperatures of flue gas and the presence of NOx, SOx as well as other impurities of the fossil fuel used (Kumar et al., 2011). However, the employment of thermophilic and elevated CO_2_ tolerant cyanobacterial species in large water reservoir experiments can solve the problem of NOx, SOx, etc. on CO_2_ sequestration from flues gas as suggested by Jansson and Northen (2010). Thermophilic cyanobacteria such as *Synechococcus lividus* and *Mastigocladus laminosus* inhabiting range from 63–64°C and 73–74°C, respectively (Miller et al., 2007). In the over all, the large body operations regarding CO_2_ sequestration from flue gas owing to the application of thermophilic cyanobacteria may be economically feasible as:

• Thermophilic cyanobacteria can efficiently assimilate significant quantity of CO_2_ from flue gas;
• Thermotolerant cyanobacterial strains may be unaffected by the NOx and SOx in flue gas;
• Use of thermophilic cyanobacteria may minimize the cost of cooling the flue gas;
• Municipal wastewater mediated nutrient supply can reduce the operation cost; and
• Freshwater and marine cyanobacterial species can be used for a broad range survival.

There are additional factors like the availability of light, pH, O_2_ removal, suitable design of the experimental systems, culture density, and the proper agitation of the systems that will affect significantly CO_2_ sequestration. Cyanobacterial CO_2_ fixation in photobioreactors has recently gained renewed interest in being the promising strategy for CO_2_ mitigation. A number of studies have been conducted during the past few decades (Hanagata et al., 1992; Maeda et al., 1995; Hirata et al., 1996a,b; Rangel-Yagui et al., 2004) related to this strategy for CO_2_ sequestration. The use of photobioreactors provides principal advantages over open-pond system, i.e., controlled environmental conditions and optimized space/volume utilization, leads to increase in cyanobacterial productivity; efficient use of land (Muhs, 2000; Ono and Cuello, 2004); higher water-use efficiency since water loss due to evaporation could be easily prevented, and improved harvesting efficiency. Also, genetically engineered cyanobacterial strains, if appropriate, could be used without disturbing the natural environment.

Through photosynthesis and calcification, cyanobacteria have the potential to capture CO_2_ from flue gas and store it precipitated as CaCO_3_/CaHCO_3_ (Mazzone et al., 2002; Lee et al., 2004). Calcium is abundant in many terrestrial, marine and lacustrine ecosystems. By using halophilic cyanobacteria, seawater or brines, for example agricultural drainage water, or saline water produced from petroleum production or geological CO_2_ injections, can serve as the potential calcium sources for the calcification process. Calcification can further be boosted by supplying calcium from gypsum (Mazzone et al., 2002) or silicate minerals, possibly in connection with biologically accelerated weathering. However, identification and characterization of cyanobacterial species that would show significant CO_2_ assimilation rates at elevated temperature and CO_2_ concentrations is still required. We have to investigate calcification at higher CO_2_ concentrations, such as in flue gas, and identify how photosynthetic machinery and light harvesting systems can be automated in cyanobacteria cultivated in open pond environment or in photobioreactors. A better understanding of the biochemical and genetic mechanisms that carry out and regulate cyanobacteria-mediated CO_2_ sequestration should put us in a position to further optimize these steps by application of advanced technique of genetic engineering.

### Cyanobacteria in Reduction of Methane Emissions

**FIGURE 6:**
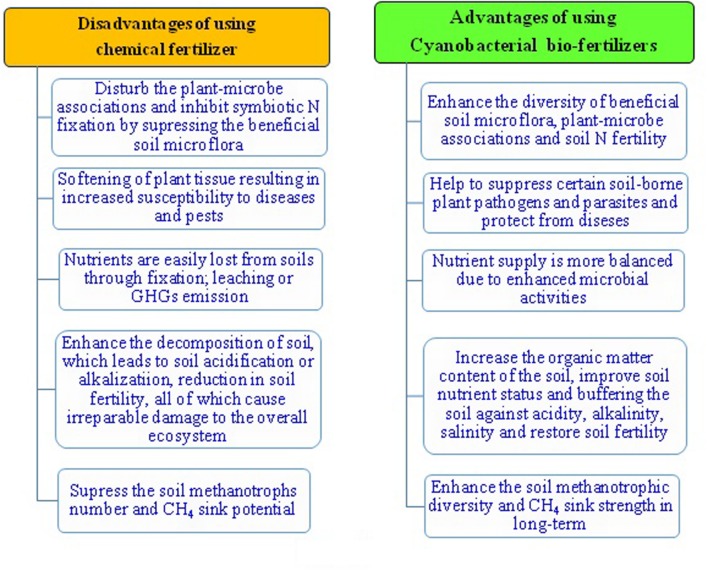
**Benefits of cyanobacterial bio-fertilizers for sustainable agriculture and mitigating the problems of CH_4_ emissions from agriculture fields**.

Methane (CH_4_) has negative consequences as it is a potent GHG with approximately 20 times the impact of CO_2_ (Singh, 2011). Anthropogenic activity accounts for the majority of global CH_4_ increase, with natural emissions accounting for the rest. Anthropogenic mediated CH_4_ emissions are due fossil fuel use, livestock farming, land filling and biomass burning. Natural sources of CH_4_ are estuaries, rivers, lakes, permafrost, gas hydrates, wetlands, oceans, wildfires, vegetation, termites, and wild animals. Flooded paddy fields are also one of the major contributors to atmospheric CH_4_ increase due to methanogenesis in anaerobic flooded paddy soils. It is assumed that with the increased human population and food requirements, greater waste generation, and greater use of fossil fuels, its concentration in the atmosphere will in all likelihood increase further. Therefore, a suitable eco-friendly and viable tool will require mitigating the problem of CH_4_. Cyanobacteria could be a big prospect to overcome the global warming problem caused by the GHGs generated from anthropogenic activities (Cuellar-Bermudez et al., 2014). Cyanobacteria may possibly minimize the emissions of CH_4_ from flooded rice soils at the levels of production, transport, and consumption. Bio-agents like methanotrophs (Tiwari et al., 2015) can play a very significant role to remove significant amount of the most potent and dangerous GHGs such as CH_4_ from the soils of various ecosystems (Singh, 2013a; Singh and Pandey, 2013; Singh and Singh, 2013a) in association with cyanobacteria. Information on interaction between cyanobacteria and methanotrophs with reference to methane flux regulation in paddy fields is completely lacking to date (Kaushik and Venkataraman, 1982). It is assumed that cyanobacteria may enhance the oxygen concentration in rhizosphere of paddy and consequently may enhance the methane uptake activity of methanotrophs. In addition, these biological agents can minimize the global warming potential from flooded paddy apart from their ability to fix the atmospheric N_2_ in the paddy soils. The O_2_, released during photosynthesis by cyanobacteria into the flooded soils, can liberate into the soil and create an aerobic environment, not friendly for CH_4_ genesis (Prasanna et al., 2002). At the same time, the O_2_ released by cyanobacteria, can augment CH_4_ oxidation by enhancing the population and activity of aerobic methane-oxidizing bacteria (methanotrophs) in flooded paddy soils. The combined application of organic amendment such as FYM and cyanobacteria can not only give the higher paddy yield, but may also contribute to production of lesser CH_4_ during paddy cultivation than the application of FYM alone (Singh et al., 2010; Singh and Pandey, 2013). Application of cyanobacteria reduces methane flux without affecting rice yields, and can be used as the practical mitigation option for minimizing the global warming potential of flooded paddy ecosystems and enhancement by N_2_ fixation (Prasanna et al., 2002). It appears that increasing the diversity of microbes (Singh, 2015a) including cyanobacteria and methanotrophs in paddy fields can be an innovative strategy to enhance crop productivity and reduce the CH_4_ emissions from the agriculture fields in the long-term (Singh and Singh, 2012; Singh, 2014). It is suggested that the application of cyanobacteria and their contributions as the N fertilizer replacement would be cost-effective, eco-friendly, and the safer means for degraded land restoration (Pandey et al., 2014; Singh, 2014, 2015b), and also to conserve the methanotrophic diversity and CH_4_ consumption in the long-term (**Figure 6**).

### Cyanobacteria as Food Supplements

Cyanobacteria as food supplements for humans are available in the market in different forms such as tablets, capsules, and liquid (Radmer, 1996). They are considered to enhance the nutritive value of pastas, snack foods, candy bars or gums, and beverages (Liang et al., 2004). They can act as the nutritional supplement or represent a source of natural food colorants (Nelis and DeLeenheer, 1991; Borowitzka, 1999; Muller-Feuga, 2000; Branen et al., 2002; Becker, 2004; Rangel-Yagui et al., 2004; Bhaskar et al., 2005; Soletto et al., 2005). The most commercial cyanobacterial strain (**Table 8**) used for human nutrition is *Spirulina* (*Arthrospira*), because of its high protein content and excellent nutritive value (Desmorieux and Decaen, 2005; Soletto et al., 2005). In many countries including Chile, Mexico, Peru, and Philippines; some cyanobacterial members such as *Spirulina, Anabaena*, and *Nostoc* are consumed as human food. *Arthrospira platensis* (*Spirulina platensis*) is grown on large scale using either raceway ponds or sophisticated photobioreactors and marketed as powder, flakes, tablets or capsules. It contains more than 60% proteins and is rich in beta-carotene, thiamine, and riboflavin, and is considered to be one of the richest sources of vitamin B_12_ (Plavsic et al., 2004; Prasanna et al., 2010). It is used as a food supplement because of its richness in nutrients and digestibility (Brown et al., 1997; Bandaranayake, 1998; Sinha et al., 1998). Kulshreshtha et al. (2008) claimed that *Spirulina* contains a wide spectrum of prophylactic and therapeutic nutrients including B-complex vitamins, minerals, proteins γ-linolenic acid and super antioxidants such as β-carotene, Vitamin E, trace elements, and a number of unexplored bioactive compounds (Nakamura et al., 1982; Garcia_Pichel et al., 1993; Bohm et al., 1995; Rimbau et al., 2001; Sinha et al., 2001; Kedar et al., 2002; Rissanen et al., 2002; Romay et al., 2003; Benedetti et al., 2004; Rajeev and Xu, 2004; Subhashini et al., 2004).

**Table 8:** Some commercial companies involved in production of cyanobacteria as food source (Courtesy of Gantar and Svircev, 2008; Priyadarshani and Rath, 2012).

S. No.	Cyanobacterial genera	Commercial company
1.	*Arthrospira* (*Spirulin*a)	Siam Algae Co. Ltd, Thailand Earthrise Nutritionals, Irvine, CA, USA Hainan Simai Pharmacy Co., China Klamath Falls Lake, Oregon, USA Lake Chad, Chad Cyanotech Corp., Kailua-Kona, HI, USA Myanmar Spirulina Factory, Myanmar
2.	*Aphanizomenon flos-aquae*	Blue green fields, USA Vision, USA

## Conclusion and Future Recommendations

It is imperative for the healthy agro-ecosystem to gain sustainability in the true sense in order that it conserves the nature and natural resources, and also maintains the complexity and diversity of the ecosystems. It supports and sustains sufficient food production for the increasing world population, ensures economic viability, and safer living for both humans as well as other livestock. Above all, it addresses the present day environmental concerns. For poor farmers (especially in developing countries), it is not quite easy to afford the costly chemical fertilizers and pesticides and also feel concerned for the environmental issues. Cyanobacteria in this context can be very effective for enriching soil organic carbon and nitrogen and enhancing phosphorus bioavailability to the plants. Cyanobacteria are excellent accumulators or degraders of various environmental contaminants such as heavy metals, pesticides, and oil containing compounds. Such ubiquitous bio-agents can also be used for capturing and storage of CO_2_ that may also lead to climate change mitigations through photosynthesis and biological calcification. They are also the ideal source of variety of bioactive compounds with marked antagonistic properties.

There is enormous scope for the development of bio-agents including cyanobacteria for sustainable agriculture which also takes care of the improvement in the nutrient status of soil and biological control of pest and diseases that may ultimately lead to reductions in the agricultural costs (Singh, 2013b; Singh and Singh, 2013b). However, it is necessary to carry out further investigations for exploitation of cyanobacteria with the futuristic goal to achieve the target of sustainable agriculture and environment. In view of the declining soil health and productivity due to increased human activities, the maintenance of environmental sustainability is the challenging task ahead. The cyanobacteria are multi-functional bio-agents for safe and eco-friendly agriculture and environmental sustainability, along with several other uses. To improve their utility in agriculture and associated sectors needs serious attention. Thus there is an urgent need to address certain key issues of exploiting cyanobacteria, the better way. Further, the application of molecular biology has improved our understanding of the effectiveness for betterment of healthy and sustainable agro-ecosystems. Since the use of cyanobacteria to produce valuable chemicals including food supplements is still little explored, there seems a long way to go. In addition to product developments, future research must address the strain improvement of useful cyanobacteria to achieve high quality food and fuel products, maintain high growth rates and survival under harsh environmental conditions. These will be the key factors to leap from laboratory studies to large-scale and profitable bio-fuel production for sustainable agriculture, ecosystem and environmental development.

The utility of cyanobacteria in sustainable agriculture and environment can be enhanced by genetic manipulations (Golden et al., 1987; Koksharova and Wolk, 2002; Huang et al., 2010; Heidorn et al., 2011). However, the application of genetic engineering to improve bio-fuel production in cyanobacteria is still in its infancy. In future, genetic and metabolic engineering of cyanobacteria are likely to play important roles in improving the economics of cyanobacteria-mediated bio-fuel production. Cyanobacteria can be genetically modified to potentially increase their growth and photosynthetic efficiency, biomass yield, lipid and carbohydrate productivity, improve temperature tolerance, and reduce photo-inhibition and photo-oxidation (Volkmann and Gorbushina, 2006; Volkmann et al., 2006). However, from lab to field condition shift will not be as easy as it has to addressed several issues such as social relevance, political lobbying and fulfillments with the regulatory norms. Besides these, problems related to cross-contamination through use of closed-photo-bioreactors as a substitute of open ponds, it is recommended to be thoroughly examined prior to execution.

## Author Contributions

JSS contributed about the role of cyanobacteria in mitigation of GHGs and overall sustainable development. AK described the role of cyanobacteria in biogas and bio-fuel production. ANR suggested about contribution of cyanobacteria in agriculture production and degraded land restoration. DPS contributed about soil nitrogen fixation and an enrichment mediated by cyanobacteria.

## Conflict of Interest Statement

The authors declare that the research was conducted in the absence of any commercial or financial relationships that could be construed as a potential conflict of interest.

## Abbreviations

BGA: blue green algae
BOD: biological oxygen demand
CCS: carbon capturing and storage
COD: chemical oxygen demand
EPS: extracellular polymeric substances
FYM: farm yard manure
GHGs: green house gases
WHC: water holding capacity
